# Nanolayer-like-shaped MgFe_2_O_4_ synthesised *via* a simple hydrothermal method and its catalytic effect on the hydrogen storage properties of MgH_2_

**DOI:** 10.1039/c8ra02168f

**Published:** 2018-04-25

**Authors:** N. A. Ali, Nurul Hayati Idris, M. F. Md Din, N. S. Mustafa, N. A. Sazelee, F. A. Halim Yap, N. N. Sulaiman, M. S. Yahya, M. Ismail

**Affiliations:** School of Ocean Engineering, Universiti Malaysia Terengganu 21030 Kuala Terengganu Malaysia mohammadismail@umt.edu.my +609-6683991 +609-6683487; Department of Electrical and Electronic Engineering, Faculty of Engineering, National Defence University of Malaysia Kem Sungai Besi Kuala Lumpur Malaysia

## Abstract

In this study, the effect of nanolayer-like-shaped MgFe_2_O_4_ that is synthesised *via* a simple hydrothermal method on the performance of MgH_2_ for hydrogen storage is studied. MgH_2_ + 10 wt% MgFe_2_O_4_ is prepared by using the ball milling method. The MgFe_2_O_4_-doped MgH_2_ sample started to release H_2_ at approximately 250 °C, 90 °C and 170 °C lower than the milled and pure MgH_2_ respectively. At 320 °C, the isothermal desorption kinetic study has shown that the doped sample has desorbed approximately 4.8 wt% H_2_ in 10 min while the milled MgH_2_ desorbed less than 1.0 wt% H_2_. For isothermal absorption kinetics, the doped sample can absorb approximately 5.5 wt% H_2_ in 10 min at 200 °C. Meanwhile, the undoped sample absorbs only 4.0 wt% H_2_ in the same condition. The activation energy of 10 wt% MgFe_2_O_4_-doped MgH_2_ composite is 99.9 kJ mol^−1^, which shows a reduction of 33.1 kJ mol^−1^ compared to the milled MgH_2_ (133.0 kJ mol^−1^). X-ray diffraction spectra display the formation of new species which are Fe and MgO after dehydrogenation, and these new species are believed to act as the real catalyst that plays a crucial role in improving the sorption performance of the MgFe_2_O_4_-doped MgH_2_ system by providing a synergetic catalytic effect.

## Abstract 2

The nanolayer-like-shaped MgFe_2_O_4_-added MgH_2_ composite showed an improvement in de/absorption properties compared to that of un-doped MgH_2_.

## Graphical abstract

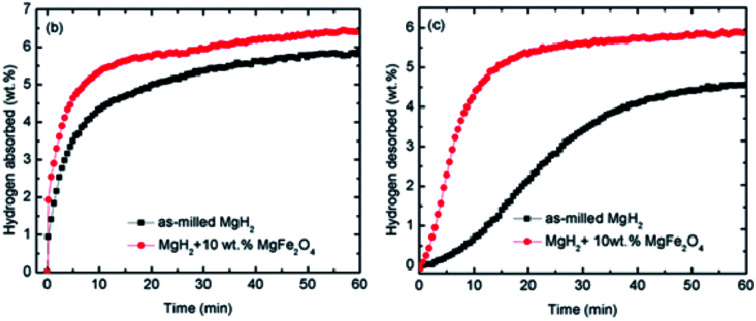

## 1. Introduction

To prepare for the future and ensure global environmental viability, energy systems have to be reliable, clean, low cost, environmentally friendly and flexible. Humanity is expected to use 40 TW of power (40 billion of kW) in the future. To satisfy this demand, different sources of renewable energy, such as hydrogen, are needed. Sustainable hydrogen is an ideal clean energy carrier because there is no carbon dioxide or other greenhouse gas emission at the end-user level. Commonly, there are 3 forms of storing hydrogen which is high-pressure gas, cryogenic liquid hydrogen in tanks (stored at 21.2 K) and as solid state hydrogen storage by either reacting with chemical compounds or absorbing. Among these approaches, solid-state hydrogen storage has higher potential for higher hydrogen density and may yield greater utility towards the practical implementation of hydrogen storage. Among the various materials for solid-state hydrogen storage, MgH_2_ considered as one of the most potential material due to its high hydrogen storage capacity (7.6 wt%), excellent reversibility and low cost.^1^ However, MgH_2_ is restricted by the decomposition temperature, which is high with slow sorption kinetics and is excessively stable thermodynamically.^2^ Many research have been conducted to overcome these disadvantages by altering the thermodynamics and improve the kinetic properties by producing nanostructures^3,4^ and utilizing catalysts such as carbon-based materials,^5,6^ metals,^7–10^ metal hydrides,^11,12^ metal oxides,^13–18^ metal halides,^19–21^ and nanosized alloys.^22–24^

Previous research has proved that catalyst based on ternary metal oxides greatly improved the hydrogen storage performance of MgH_2_.^15,25–35^ Zhang *et al.*^25^ demonstrate that ferrite nanoparticles (MnFe_2_O_4_, ZnFe_2_O_4_, Mn_0.5_Zn_0.5_Fe_2_O_4_ and CoFe_2_O_4_) can greatly lower the decomposition temperature of MgH_2_. CoFe_2_O_4_ provides the best catalytic effect compared with other ferrites. New by-products are found after the dehydrogenation process, and its phase shows a great catalytic effect on the properties of hydrogen storage of MgH_2_. Furthermore, Li *et al.*^26^ shows a significant improvement in the desorption performance of MgH_2_ when catalysed with MnFe_2_O_4_. X-ray photoelectron spectroscopy and X-ray diffraction (XRD) tests show that Fe_0.872_O and Mg_2_MnO_4_ phases take part as significant role in enhancing the dehydriding performance of MgH_2_. Meanwhile, we showed in our previous study that MnFe_2_O_4_ synthesised *via* a simple hydrothermal method provides a remarkable effect in improving the hydrogen storage performance of MgH_2_.^27^ Interestingly, our result showed that Fe metal formed after dehydrogenation instead of Fe_0.872_O species, as claimed by Li *et al.*^26^ This variation paved the way for the debate on how ternary metal oxides, particularly ferrites, work as catalysts in improving the hydrogen sorption performance of MgH_2_. Moreover, the difference in the synthesis method of the catalysts may also provide a different effect in the catalytic role.

Inspired by the role of active species that formed during the heating process in the MgH_2_-ternary metal oxides catalyst system, it is quite interesting to investigate the use of other ferrites (*e.g.* MgFe_2_O_4_) as catalysts to improve the hydrogen sorption performance of MgH_2_. Therefore, in this work, MgFe_2_O_4_ was synthesised by using a simple hydrothermal method, and its catalytic effects on the hydrogen sorption performance of MgH_2_ were systematically studied. To the best of authors' knowledge, this paper is the first to study the hydrogen sorption performance of MgH_2_ catalysed with MgFe_2_O_4_. The possible catalysis mechanisms of MgFe_2_O_4_ in the sorption performances of MgH_2_ are also discussed in this paper.

## 2. Experimental details

The nanolayer-like-shaped MgFe_2_O_4_ was synthesised *via* a hydrothermal method. In a typical synthesis, a stoichiometric amount of Mg(NO_3_)_2_·6H_2_O (Sigma-Aldrich) and Fe(NO_3_)_3_·9H_2_O (Sigma-Aldrich) were dissolved in 50 ml distilled water. A total of 10 ml of H_4_N_2_·H_2_O (Sigma-Aldrich) was added dropwise to the above solution to attain the resultant pH of >9. The mixture was then transferred into a sealed Teflon lined stainless-steel autoclave (125 ml capacity) and heated for 12 h at 180 °C. The final product was washed several times with deionised water and dried overnight at 60 °C under vacuum. A total of 10 wt% of as-prepared MgFe_2_O_4_ was mixed with 300 mg of MgH_2_ (95% pure; Sigma-Aldrich) and undergo intensive ball milling for 1 h in a planetary ball mill at the rate of 400 rpm. For comparison, pure MgH_2_, and MgH_2_ added with 10 wt% Fe (Alfa Aesar) and 10 wt% MgO (R&M Chemicals), respectively were also prepared under the same conditions. All preparations, including loading and weighing, were conducted in an argon atmosphere glove box (MBraun Unilab).

The onset decomposition temperature and sorption kinetic measurement for doped and undoped samples were characterised by using Sievert-type pressure–composition–temperature apparatus (Advanced Materials Corporation). For onset decomposition temperature measurement, the samples were heated from room temperature to 450 °C at a heating rate of 5 °C min^−1^ in vacuum chamber. Meanwhile, the sorption kinetics was conducted under 1.0 atm at 320 °C for desorption kinetic measurement and under 33.0 atm at 200 °C for absorption kinetic measurement. The thermal properties of the doped and undoped samples were performed using differential scanning calorimeter (DSC)/thermogravimetric analysis from Metler Toledo. With a flow of 50 ml min^−1^ argon, the samples were heated with 15, 20, 25 and 30 °C min^−1^ heating rate from 25 °C to 500 °C.

The phase composition of the samples was analysed by XRD *via* a Rigaku MiniFlex X-ray diffraction apparatus equipped with Cu Kα radiation. Data were collected in the 2*θ* range 20° to 80° at 2° min^−1^. The morphologies of the samples were observed by scanning electron microscopy (SEM) (JEOL JSM-6350LA). Fourier transform infrared (FTIR) spectrometry was recorded on an IR Shimadzu Tracer-100 between 400 and 2000 cm^−1^. Raman spectra were recorded on Renishaw Raman spectroscopy (532 nm radiation) extended with 0.1% power laser measurement at room temperature.

## 3. Results and discussion

**Fig. 1:**
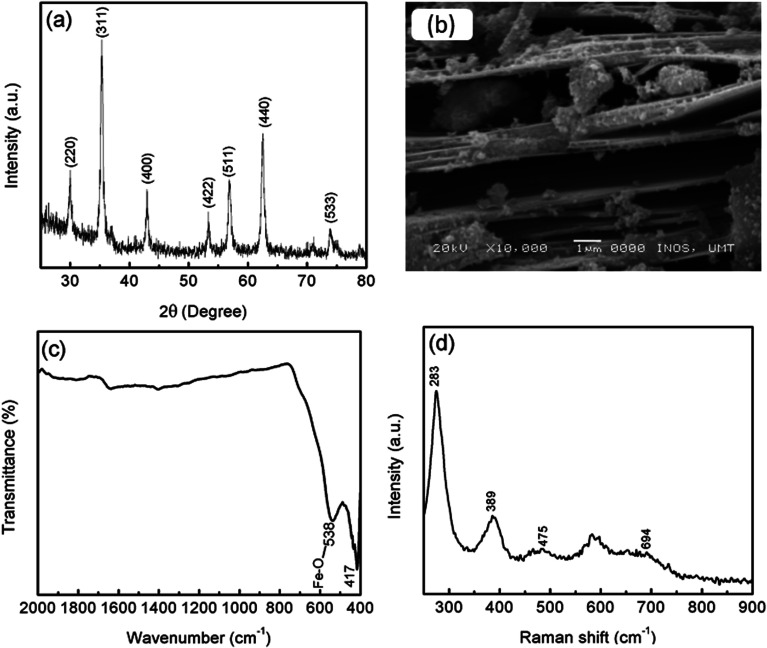
(a) XRD pattern, (b) SEM image, (c) FTIR spectra and (d) Raman spectra of MgFe_2_O_4_.

Before milling with MgH_2_, the phase structure of MgFe_2_O_4_ was confirmed by XRD, as shown in Fig. 1(a). The crystallographic planes of (220), (311), (400), (422), (511), (440) and (533) correspond to the diffraction peaks at 2*θ* of 30.1°, 35.4°, 43.1°, 53.5°, 57.0°, 62.6° and 74.1°, respectively. This result is in good agreement with the standard cubic spinel structure (JCPDS 71-1232). No other peaks were detected in the sample. The average crystallite size of MgFe_2_O_4_ was approximately 19 nm, as determined by using Scherrer's formula:

1 *L* = k*λ*/*B* cos *θ*,

where *L* is the average crystallite size (nm), *θ* is the angle of diffraction, *k* is Scherrer's constant (*k* = 0.94), *λ* is the X-ray wavelength (0.15405 nm) and *B* is the full width at half maximum of the diffraction peak in radian (FWHM). The SEM image (Fig. 1(b)) reveals that the MgFe_2_O_4_ forms a large layer with a nanosized thickness. From the FTIR spectrum (Fig. 1(c)), two typical peaks of MgFe_2_O_4_ were observed at the low wavenumber, thus indicating the formation of spinel ferrite structure.^36,37^ The peak at 417 cm^−1^ can be ascribed to the Fe–O vibration in the octahedral site, and the peak at 538 cm^−1^ can be assigned to the Fe–O vibration in the tetrahedral and octahedral sites. Furthermore, the Raman peaks (Fig. 1(d)) at 475 and 694 cm^−1^ can be assigned to the typical characteristic peaks of MgFe_2_O_4_,^38^ whereas the peak at 283 cm^−1^ corresponding to the stretching vibration of the Mg–O chemical bond.^39^ The XRD, FTIR and Raman spectroscopy results confirm that pure MgFe_2_O_4_ was successfully synthesised by the hydrothermal method.

**Fig. 2:**
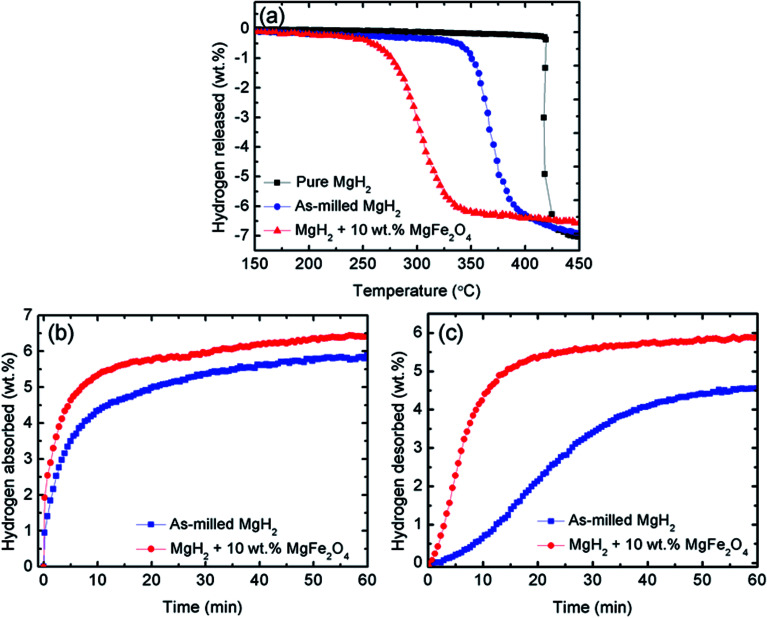
(a) Decomposition temperature profile of pure and milled MgH_2_, and 10 wt% MgFe_2_O_4_-doped MgH_2_ sample, (b) measurement of isothermal absorption kinetics for the milled MgH_2_ and 10 wt% MgFe_2_O_4_-doped MgH_2_ sample at 200 °C under 33.0 atm H_2_ pressures, and (c) measurement of isothermal desorption kinetics for the milled MgH_2_ and 10 wt% MgFe_2_O_4_-doped MgH_2_ sample at 320 °C under 1.0 atm.

Fig. 2(a) shows the onset decomposition temperature results for the pure MgH_2_, milled MgH_2_ and MgH_2_ with 10 wt% MgFe_2_O_4_. Before milling, pure MgH_2_ started to desorb hydrogen at approximately 420 °C. The total amount of hydrogen desorbed is approximately 7.0 wt%. After milling for 1 h, the onset decomposition temperature of MgH_2_ was decreased to approximately 340 °C. This phenomenon demonstrate that the sorption performance of MgH_2_ also influenced by the milling process. From the curve, it can be seen that after milling, the amount of hydrogen desorb of MgH_2_ slightly decreases. This might be ascribed to the hydrogen released from MgH_2_ during the milling process. After doping with 10 wt% of MgFe_2_O_4_, it is clear that the onset decomposition temperature of the MgH_2_ was dramatically reduced to 250 °C, 90 °C and 170 °C lower than that for the milled and pure MgH_2_, respectively. However, the hydrogen desorption capacity decrease slightly to approximately 6.5 wt% because the dopant used in this study, namely, MgFe_2_O_4_, does not contain hydrogen.^40^ From Fig. 2(a), it can be concluded that MgFe_2_O_4_ additive plays a positive role in decreasing the decomposition temperature of MgH_2_.

To further examine the sorption properties of the MgFe_2_O_4_-doped MgH_2_ sample, the isothermal absorption kinetic was studied. The amount of hydrogen absorbed from the milled MgH_2_ and the MgFe_2_O_4_-doped MgH_2_ sample was measured under 33.0 atm H_2_ and at constant temperature of 200 °C, as shown in Fig. 2(b). The MgFe_2_O_4_-doped MgH_2_ sample shows better absorption kinetics than undoped MgH_2_. For the MgFe_2_O_4_-doped MgH_2_ sample, the amount of 5.5 wt% H_2_ was absorbed in 10 min, whereas the milled MgH_2_ only absorbed approximately 4.0 wt% H_2_ within the same time. From the result, it clearly shows that the addition of MgFe_2_O_4_ enhanced the rehydrogenation kinetics of MgH_2_.

For further studies on the catalytic effect of MgFe_2_O_4_ on the sorption kinetic of MgH_2_, isothermal desorption kinetic was performed under 1.0 atm at 320 °C. As shown in Fig. 2(c), the MgFe_2_O_4_-doped MgH_2_ sample shows significant enhancement compared with the milled MgH_2_. The results shows that the undoped MgH_2_ released less than 1.0 wt% H_2_ after 10 min, whereas the doped sample can released approximately 4.8 wt% H_2_ under the same condition. In contrast, it can be seen that MgFe_2_O_4_ also plays a significant role in enhancing the dehydrogenation kinetic of MgH_2_.

**Fig. 3:**
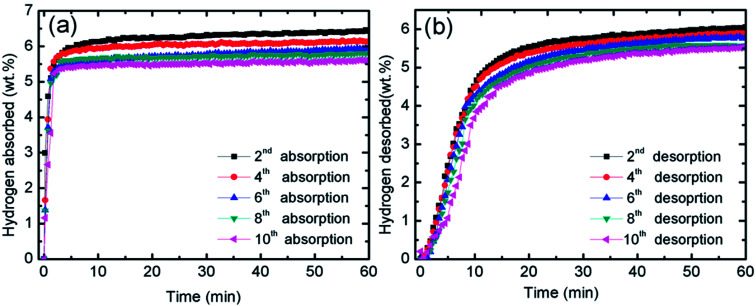
(a) Measurement of isothermal absorption kinetics for the 10 wt% MgFe_2_O_4_-doped MgH_2_ sample in the 2^nd^, 4^th^, 6^th^, 8^th^ and 10^th^ cycle at 320 °C under 33.0 atm H_2_ pressures and (b) measurement of isothermal desorption kinetics for the 10 wt% MgFe_2_O_4_-doped MgH_2_ sample in the 2^nd^, 4^th^, 6^th^, 8^th^ and 10^th^ cycle at 320 °C under 1.0 atm.

The catalytic effect of MgFe_2_O_4_ was further studied with the cycling performances of MgFe_2_O_4_-doped MgH_2_ system. Fig. 3(a) presents the isothermal absorption kinetics of the 10 wt% MgFe_2_O_4_ doped with MgH_2_ at 320 °C under a hydrogen pressure of 33.0 atm over 10 cycles. From the result, it can be seen that after the ten cycles, the absorption kinetics show a small reduction in the hydrogen capacity. After completing the 10^th^ cycle, the system is able to absorb 5.6 wt% of hydrogen in 60 minutes. The result shows that the doped system displays good absorption properties even after 10 cycles. As for the desorption kinetics, Fig. 3(b) shows the isothermal desorption kinetics for 10 cycles that was carried out at 320 °C and under 1.0 atm of pressure. Like the absorption kinetics, a small hydrogen capacity degradation is shown after completing the 10^th^ cycle. The doped system possesses a good performance after completing the 10^th^ cycle as it is able to desorb about 5.5 wt% of hydrogen within 60 minutes. These results demonstrated that MgFe_2_O_4_ plays a vital catalytic role for the cycle life of MgH_2_.

**Fig. 4:**
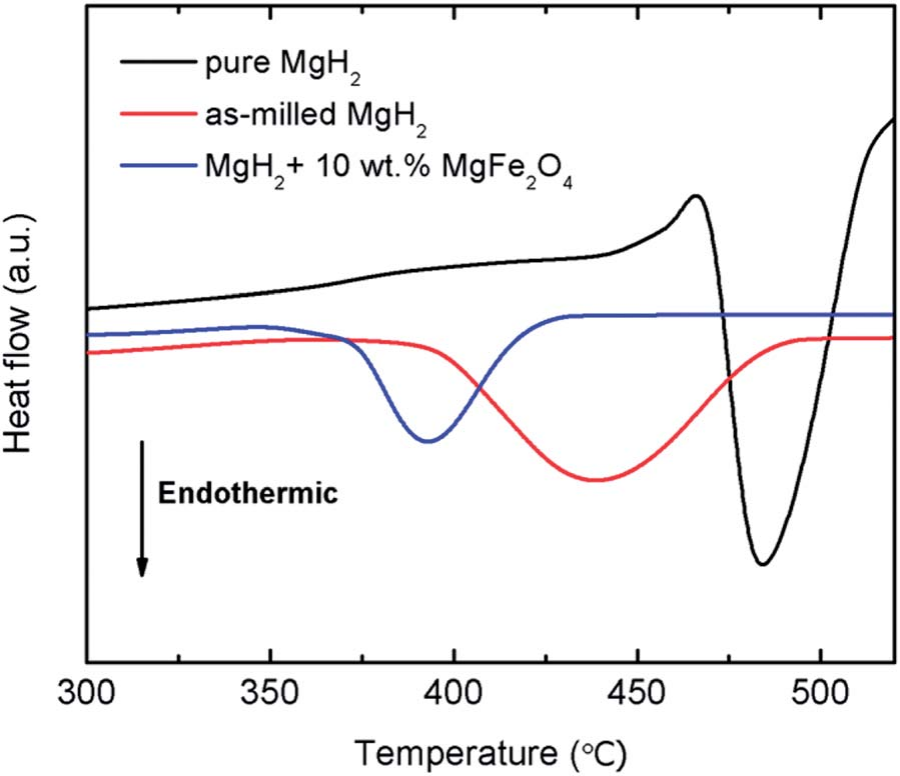
DSC curves of pure MgH_2_, milled MgH_2_, and 10 wt% MgFe_2_O_4_-doped MgH_2_.

The thermal properties of the 10 wt% MgFe_2_O_4_-doped MgH_2_ and undoped MgH_2_ sample were further studied by DSC at heating rate of 30 °C min^−1^ and under a flow of 50 ml min^−1^ argon (Fig. 4). Obviously, the DSC trace for the pure MgH_2_ showed one endothermic peak at approximately 482.9 °C. This strong endothermic peak related to the released of hydrogen from the MgH_2_. Similar to the pure MgH_2_, DSC traces of the milled MgH_2_ and MgFe_2_O_4_-doped MgH_2_ showed only one strong endothermic peak at 438.8 °C and 393.3 °C respectively. The peaks correlated to the decomposition of MgH_2_ but at lower temperatures.

The improvement in desorption behaviour is correlated with the kinetic barrier of the hydrogen desorbed from the MgH_2_. By doping MgH_2_ with MgFe_2_O_4_, low value of activation energy for released hydrogen is obtained. Kissinger analysis^41^ (eqn (2)) was conducted to determine the activation energy of doped and undoped MgH_2_ samples.

**Fig. 5:**
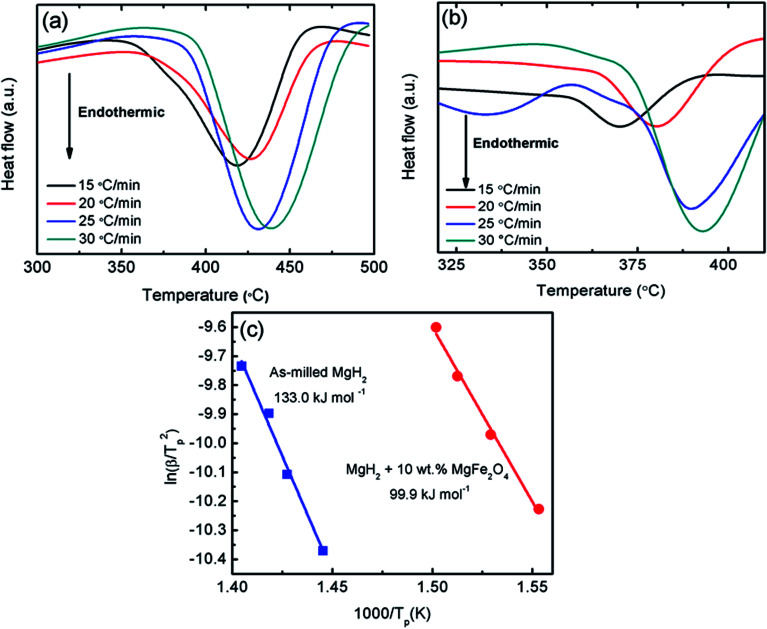
DSC curves at heating rates of 15, 20, 25, and 30 °C min^−1^ for (a) milled MgH_2_, (b) MgH_2_ +10 wt% MgFe_2_O_4_ and (c) the Kissinger plot of decomposition for 10 wt% MgFe_2_O_4_-doped MgH_2_ composite and milled MgH_2_.

2 ln[*β*/*T*_p_^2^] = −*E*_A_/*RT*_p_ + *A*,

where *β* is the heating rate, *E*_A_ is the activation energy, *R* is the gas constant, *T*_p_ is the peak temperature of DSC curves and A is the linear constant. Fig. 5 illustrates the curves of DSC for the milled MgH_2_ and MgH_2_ doping with 10 wt% MgFe_2_O_4_ samples at heating rates of 15, 20, 25, and 30 °C min^−1^. From the Kissinger plot of the DSC data (Fig. 5(c)), it can be perceived that the activation energy, *E*_A_, for the MgFe_2_O_4_-doped MgH_2_ composite is 99.9 kJ mol^−1^, which is decrease 33.1 kJ mol^−1^ compared with the milled MgH_2_ (133.0 kJ mol^−1^). The result indicates that addition of MgFe_2_O_4_ reduces the decomposition activation energy and boost the desorption performances of MgH_2_.

**Fig. 6:**
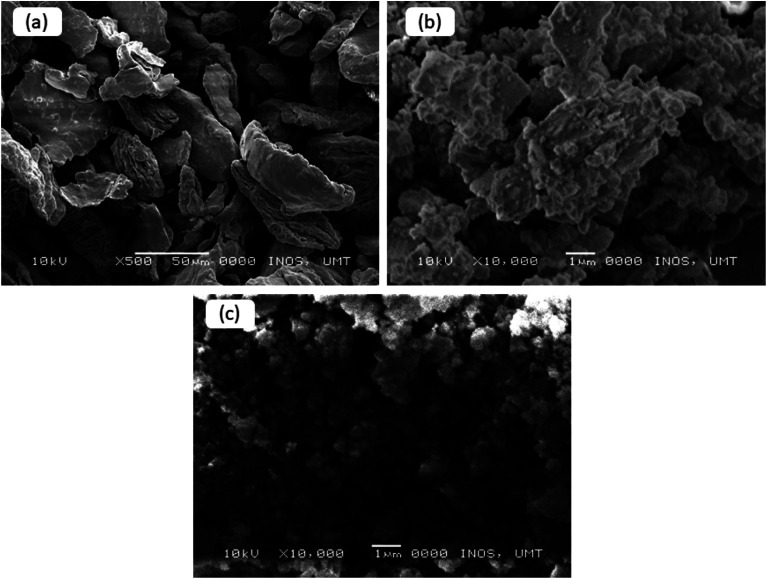
SEM images for the pure MgH_2_ (a), milled MgH_2_ (b) and MgH_2_ + 10 wt% MgFe_2_O_4_ (c).

Fig. 6 presents the microstructures of the pure and milled MgH_2_, and MgFe_2_O_4_-doped MgH_2_. From the images, it can be seen clearly that the particle size of the pure MgH_2_ is around 50–100 μm (Fig. 6(a)). Fig. 6(b) shows the image of the MgH_2_ after 1 h ball milling. The size of the milled MgH_2_ was decreased dramatically compared to the pure MgH_2_. However, the image shows agglomeration and inconsistent particle sizes. Fig. 6(c) shows that the particle size of 10 wt% MgFe_2_O_4_-doped MgH_2_ was the smallest and had less agglomeration than the pure and milled MgH_2_. Smallest particle size gives a larger region of contact to the MgH_2_, thus resulting in the higher rate of reaction of MgH_2_.

**Fig. 7:**
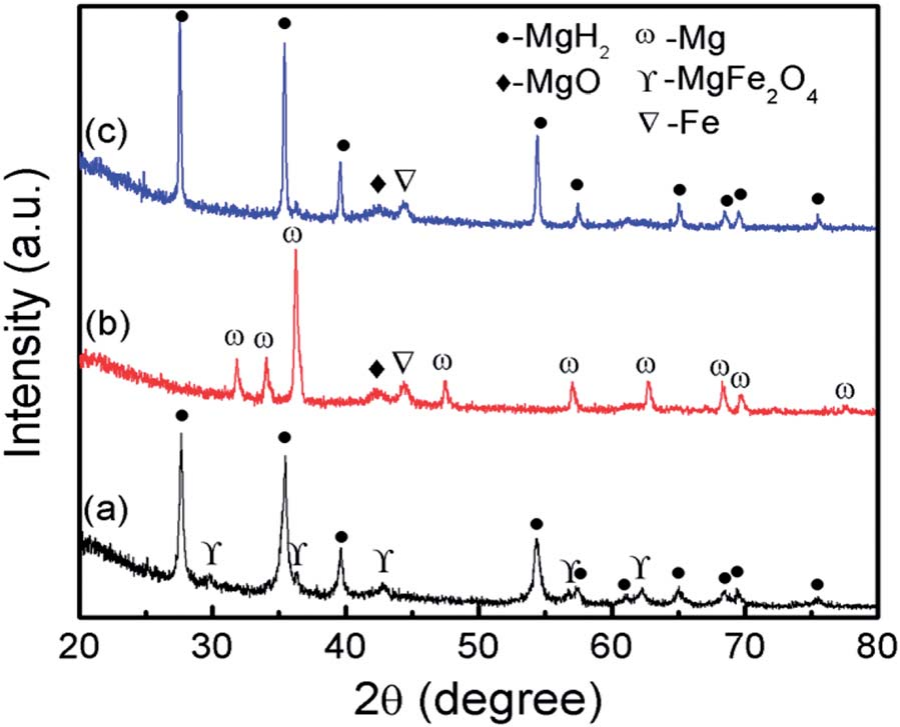
XRD spectra of the MgH_2_ + 10 wt% MgFe_2_O_4_ (a) after ball milling for 1 h, (b) after desorption under 1.0 atm at 450 °C and (c) after absorption under 33.0 atm at 320 °C.

To investigate the phase structure, XRD measurement was performed on the 10 wt% MgFe_2_O_4_-doped MgH_2_ sample, as shown in Fig. 7. From Fig. 7(a), it can be observed that the MgH_2_ and MgFe_2_O_4_ phases are present in the as-milled MgFe_2_O_4_-doped MgH_2_ sample. No additional peaks were found from the spectra. After dehydrogenation at 450 °C (Fig. 7(b)), the XRD pattern showed that the MgH_2_ was completely dehydrogenated to Mg. This result demonstrates that the decomposition of MgH_2_ was completed after heating for up to 450 °C. Furthermore, a small peak of MgO and Fe formed after the desorption process, thus demonstrate that the partial reaction of MgH_2_ with MgFe_2_O_4_ may occur during the heating process as follows:

3 4MgH_2_ + MgFe_2_O_4_ → Mg + 4MgO + 2Fe + 4H_2_

The standard Gibbs Free energy, 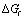, of MgH_2_, MgFe_2_O_4_ and MgO are −35.9824, −1317.1232 and −569.024 kJ mol^−1^, respectively.^42^ The total change ΔG correlated with the reaction in eqn (3) is −815.0168 kJ mol^−1^. These values can confirm the possibility of the reaction in eqn (3) from thermodynamic potentials. Fig. 7(c) shows the XRD patterns for the rehydrogenated sample under 33.0 atm H_2_ at 320 °C. The result illustrates that the phase of Mg was fully converted into MgH_2_. Furthermore, the peak of Fe and MgO still remained after undergo rehydrogenation.

**Fig. 8:**
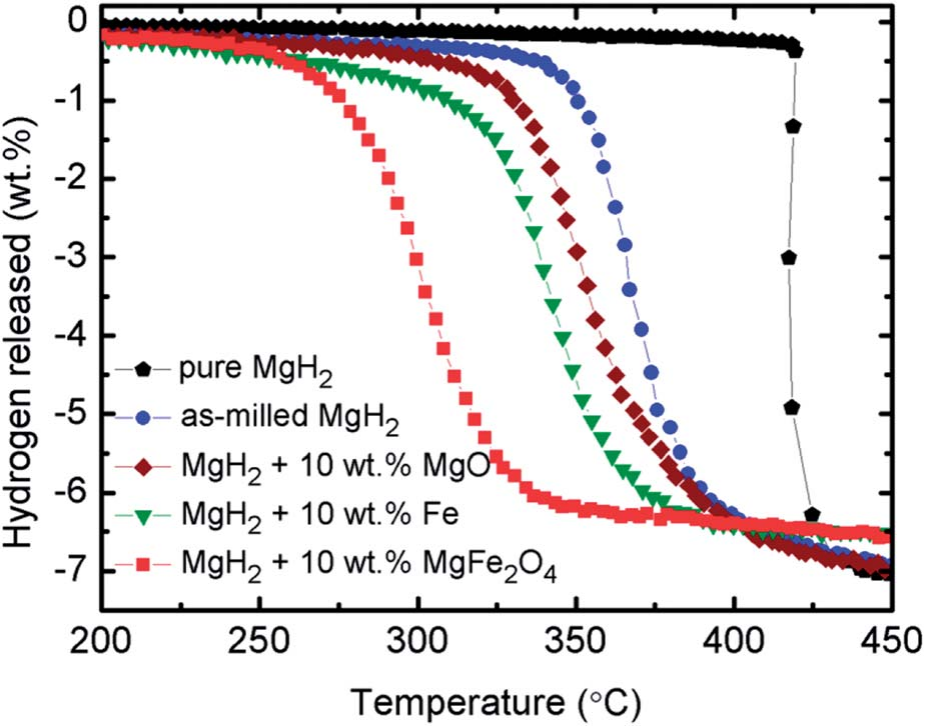
Decomposition temperature profile of pure and milled MgH_2_, 10 wt% MgO-doped MgH_2_, 10 wt% Fe-doped MgH_2_ and 10 wt% MgFe_2_O_4_-doped MgH_2_ sample.

From the above analyses, the improvements in the sorption properties of MgH_2_ doped with 10 wt% MgFe_2_O_4_ may be resulted from the formations of Fe and MgO. Fe and MgO may act as the real catalysts that play a vital role in the improvements of MgH_2_ sorptions. Therefore, to verify the effect of Fe and MgO on MgH_2_, samples of 10 wt% MgO-doped MgH_2_ and 10 wt% Fe-doped MgH_2_ were prepared and the TPD profiles for the dehydrogenations were shown as in Fig. 8. It is clearly seen that the decomposition temperature of MgH_2_ are reduced with the addition of MgO or Fe as compared to the pure and milled MgH_2_. However, the performance of these MgO and Fe are not significant as the sample of 10 wt% of MgFe_2_O_4_ doped with MgH_2_. This demonstrated that the *in situ* generated MgO and Fe from the reaction of MgH_2_ + 10 wt% of MgFe_2_O_4_ may play a significant role that introduce a synergetic catalytic effect that cause a significant improvement on the dehydrogenation performances of MgH_2_ doped with 10 wt% of MgFe_2_O_4_. In addition, the *in situ* formed Fe and MgO in the MgH_2_ + 10 wt% of MgFe_2_O_4_ sample are speculated to have a higher degree of dispersion and more compact phase segregation as compared to the milled MgH_2_ + 10 wt% Fe and milled MgH_2_ + 10 wt% MgO. This would be likely to lead the improvement of the sorption kinetics.

From the result obtained, we postulate that formation of fresh and fine MgO and Fe species which resulted from the reaction between MgH_2_ and MgFe_2_O_4_ during the dehydrogenation process may play significant role in improving the sorption performances of MgH_2_. Numerous studies have claimed that the newly active species formed during the de/absorption process may play as a real catalyst in the enhancement of MgH_2_ sorption.^43,44^ Many works have proven that Fe is an excellent catalyst for sorption performance in MgH_2_.^7–9^ It is believed that the presence of fresh and fine Fe could interact with H_2_ molecules, thus possibly leading to the dissociation of H_2_ molecules and the improvement of the de/rehydrogenation kinetic. Furthermore, previous studies have shown that the sorption performance in MgH_2_ can be enhanced with the addition of MgO. Ares-Fernández and Aguey-Zinsou^45^ claimed that the addition of MgO to MgH_2_ during the milling process led to an enhancement of sorption kinetics because of the high electronegativity MgO. In another study, the same group also claimed that during the milling process, MgO may act as a process control agent that can lead to the reduction of the particle agglomeration of MgH_2_ by an optimal breakage rate, thus aiding the high stability of these particles and evading the use of cold welding.^46^ Shan *et al.*^15^ also revealed that MgO has a great catalytic effect on the MgH_2_ sorption performance. Their study showed that during the heating process in CoFe_2_O_4_-doped MgH_2_ composite system, MgO is formed. The catalytic effect of MgO could work together with the catalytic role of the Fe metal to create a synergetic effect. Therefore, the *in situ* active species of Fe and MgO may actually act as real catalysts and further enhance the hydrogen sorption performance of MgH_2_. However, further studies are needed to elucidate more details on the exact role of MgFe_2_O_4_ addition in MgH_2_.

## 4. Conclusion

In this study, nanolayer-like-shaped MgFe_2_O_4_ was successfully synthesised through a rapid, simple hydrothermal method. The addition of 10 wt% as-synthesised MgFe_2_O_4_ to MgH_2_ reduces the onset decomposition temperature and enhances sorption kinetics. The MgFe_2_O_4_-doped MgH_2_ sample has started to release H_2_ at approximately 250 °C, 90 °C and 170 °C lower than milled and pure MgH_2_ respectively. In a duration of 10 min, the isothermal desorption kinetic study showed that the doped sample can release approximately 4.8 wt% H_2_ at 320 °C while the milled MgH_2_ only desorbed less than 1.0 wt% H_2_ under the same condition. For isothermal absorption kinetics, the doped sample can absorb approximately 5.5 wt% H_2_ in 10 min at 200 °C. By contrast, the milled MgH_2_ sample absorbed only 4.0 wt% H_2_ in the same condition. From the Kissinger analysis, the apparent activation energies, *E*_A_, for the MgFe_2_O_4_-doped MgH_2_ sample were calculated to be 99.9 kJ mol^−1^, which is decreased by 33.1 kJ mol^−1^ compared with the milled MgH_2_ (133.0 kJ mol^−1^). The XRD exploration displays the formation of new species of Fe and MgO after the dehydrogenation process, and these species remained unchanged after rehydrogenation. It is believed that the new species of Fe and MgO play a synergistic role in enhancing the hydrogen storage performances of MgH_2_.

## Conflicts of interest

There are no conflicts to declare.

## Supplementary Material
